# Self-perceived cognitive impairments in psychosis ultra-high risk individuals: associations with objective cognitive deficits and functioning

**DOI:** 10.1038/s41537-020-00124-1

**Published:** 2020-11-13

**Authors:** Louise Birkedal Glenthøj, Lise Mariegaard, Tina Dam Kristensen, Christina Wenneberg, Alice Medalia, Merete Nordentoft

**Affiliations:** 1 Copenhagen Research Centre for Mental Health (CORE), Copenhagen University Hospital, DK-2900 Hellerup, Denmark; 2 Centre for Clinical Intervention and Neuropsychiatric Schizophrenia Research, CINS, DK-2600 Glostrup, Denmark; 3 Columbia University Irving Medical Center, New York, NY 10032 USA

**Keywords:** Psychosis, Schizophrenia

## Abstract

There is a scarcity of evidence on subjectively reported cognitive difficulties in individuals at ultra-high risk (UHR) for psychosis and whether these self-perceived cognitive difficulties may relate to objective cognitive deficits, psychopathology, functioning, and adherence to cognitive remediation (CR). Secondary, exploratory analyses to a randomized, clinical trial were conducted with 52 UHR individuals receiving a CR intervention. Participants completed the Measure of Insight into Cognition—Self Report (MIC-SR), a measure of daily life cognitive difficulties within the domains of attention, memory, and executive functions along with measures of neuropsychological test performance, psychopathology, functioning, and quality of life. Our study found participants with and without objectively defined cognitive deficits reported self-perceived cognitive deficits of the same magnitude. No significant relationship was revealed between self-perceived and objectively measured neurocognitive deficits. Self-perceived cognitive deficits associated with attenuated psychotic symptoms, overall functioning, and quality of life, but not with adherence to, or neurocognitive benefits from, a CR intervention. Our findings indicate that UHR individuals may overestimate their cognitive difficulties, and higher levels of self-perceived cognitive deficits may relate to poor functioning. If replicated, this warrants a need for both subjective and objective cognitive assessment in at-risk populations as this may guide psychoeducational approaches and pro-functional interventions. Self-perceived cognitive impairments do not seem to directly influence CR adherence and outcome in UHR states. Further studies are needed on potential mediator between self-perceived cognitive deficits and functioning and quality of life.

## Introduction

Individuals at ultra-high risk (UHR) for psychosis are characterized by persistent cognitive deficits within both global and specific cognitive domains^1–6^. Meta-analytical evidence has revealed moderate overall impairments in neurocognition in UHR compared to healthy controls (Hedges’ *g* = −0.344)^7^. Additionally, the severity of neurocognitive impairments are found to be intermediate between that of patients with schizophrenia and healthy controls^3,7^. Neurocognitive deficits in UHR have been implicated in psychosis development^4,8^ and poor functional outcome^9,10^.

As performance-based cognitive testing is the preferred assessment measure employed in psychosis research, few studies have elucidated on patient’s self-perceived cognitive impairments: in general, the studies do not find an association between objective and subjective neurocognitive deficits in patients with established psychosis^11–14^. To the best of our knowledge, no previous study has, however, investigated whether this discrepancy between objective and subjective cognitive deficits also apply to the UHR state of psychosis.

Cognitive remediation is one of the most promising interventions to alleviate cognitive deficits, with proven effectiveness in first-episode and more chronic states of psychosis with small to medium effect sizes on cognitive and functional outcomes^15–21^. The existing, albeit small, literature does not indicate awareness of cognitive deficits is a pre-requisite for positive outcome of a cognitive remediation intervention in schizophrenia^13,14^. While considerably less studied, preliminary data indicate efficacy of cognitive remediation in the UHR state of psychosis^22^, but it remains, however, unclear whether self-perceived cognitive deficits may influence adherence to cognitive remediation in the UHR population. That is, whether higher levels of subjectively reported cognitive deficits will relate to UHR individuals adhering to and engaging in a cognitive remediation intervention.

By using data from the hitherto largest randomized, clinical trial (FOCUS trial)^23^ on cognitive remediation in the UHR population, we conducted secondary, exploratory analyses aimed at investigating the relationship between self-perceived neurocognitive deficits and objective cognitive performance, psychopathology, functioning, and adherence to a cognitive remediation intervention. The study addressed the following questions: (1) Is there a relationship between subjective and objective cognitive deficits in UHR individuals? (2) Is there an association between subjectively reported cognitive deficits and clinical symptoms and functioning/quality of life? (3) Do self-perceived cognitive deficits associate with adherence to and benefit from a cognitive remediation intervention?

## Results

Out of the 73 UHR individuals randomized to the cognitive remediation interventions, 52 (71%) completed the MIC-SR. Reasons for not completing the MIC-SR were: drop-out before starting the cognitive remediation intervention (*n* = 8), or lack of time to complete MIC-SR (*n* = 13). Analyses revealed that the participants completing the MIC-SR did not differ from the ones not completing the MIC-SR on any of the demographic or clinical variables. Twenty-nine (55.8%) of the participants were females, and they had a mean age of 23.98 (SD 4.6) and an average of 14.29 (SD 2.4) years of education. The participants mainly fulfilled the high-risk criteria of APS (*N* = 40, 76.9 %) (demographic and baseline measures are depicted in Table 1). Of the 52 UHR participants completing the MIC-SR, 32 (61%) showed impaired objectively measured cognition, corresponding to a BACS z-score of ≤−1.35, which has been reported to indicate borderline to impaired cognition in the general population^12^.

**Table 1:** Baseline sample demographics for 52 ultra-high risk individuals receiving cognitive remediation.

Variable	*N* (%)
Female	29 (55.77)
CAARMS status	
APS	40 (76.92)
BLIPS	0 (0)
Trait/state	1 (1.92)
APS + trait/state	10 (19.23)
APS + BLIPS	1 (1.92)

	Mean (SD)
Age	23.98 (4.57)
Years of education	14.29 (2.41)
Estimated IQ	103.54 (11.81)
MIC-SR total	21.23 (7.62)
MIC-SR average frequency	1.77 (0.64)
BACS composite	−1.04 (1.17)
CAARMS composite	49.33 (13.19)
SANS	1.51 (0.85)
MADRS	15.33 (7.28)
PSP	56.67 (10.51)
AQoL-8D	0.44 (0.14)

*CAARMS* comprehensive assessment of at-risk mental states, *APS* attenuated psychotic symptom, *BLIPS* brief limited intermittent psychotic symptom, MIC-SR measure of insight into cognition—self report, *BACS* brief assessment of cognition in schizophrenia, *CAARMS* comprehensive assessment of at-risk mental states, *SANS* scale for the assessment of negative symptoms, *MADRS* Montgomery-Åsberg Depression Rating Scale, *PSP* Personal and Social Performance Scale, *AQoL-9D* assessment of quality of life.

The participants had a mean MIC-SR total score of 21.2 (7.6) and a MIC-SR average frequency score of 1.9 (.6) indicating that the UHR individuals reported experiencing cognitive impairments more than once a week. Participants displaying objective cognitive deficits had a mean MIC-SR total score of 19.9 (SD 7.7) and a MIC-SR average frequency score of 1.9 (SD .60). Participants not displaying cognitive deficits had a mean MIC-SR total score of 23.4 (SD 7.1) and a MIC-SR average frequency of 1.7 (SD = .6).

Participants attended an average of 10.9 (SD = 7.6) out of the total 20 sessions of neuro- and social cognitive remediation and had an average of 11.9 (SD = 16.4) h of neurocognitive training (including both group- and homebased training).

### Relationship between subjective and objective cognitive deficits

The one-way ANOVA revealed that the 32 UHR individuals exhibiting objective cognitive deficits did not differ from the 20 participants not displaying objective cognitive deficits on the MIC-SR total score (*p* = 0.11). Neither did the Spearman’s correlational analyses reveal a significant baseline correlation between the MIC-SR total score and the BACS composite score (rho = −0.12, *p* = 0.39).

Additional analyses revealed that the MIC-SR total score did not significantly relate to estimated full scale IQ, verbal IQ, performance IQ, or years of education. Estimated IQ was, however, significantly related to the BACS composite score.

### Association between self-perceived cognitive deficits and clinical symptoms, functioning and quality of life

Regression analyses revealed baseline MIC-SR to associate with the baseline measures of CAARMS composite, and the PSP and AQoL-8D, but not with negative or depressive symptoms (Table 2). That is higher scores on the self-perceived cognitive impairments measure associated with greater severity of attenuated psychotic symptoms and lower functioning. The MIC-SR explained 10%, 7% and 22% of the variance on the CAARMS, PSP and AQoL-8D, respectively.

**Table 2:** Linear regression analyses of Measure of Insight into Cognition—Self Report (MIC-SR) total score associating with clinical symptoms and functioning in UHR individuals (*N* = 52).

	Univariable			
	*β* [95% CI]	*t*	*p*	*R*^2^
Symptoms				
CAARMS	0.602 [0.140 to 1.063]	2.619	0.012	0.103
SANS	0.004 [−0.028 to 0.035]	0.240	0.811	
MADRS	0.224 [−0.040 to 0.488]	1.706	0.094	
Functioning				
PSP	−0.410 [−0.794 to −0.027]	−2.149	0.037	0.067
AQoL-8D	−0.009 [−0.013 to −0.004]	0–3.904	*p* < 0.001	0.218

*R*^2^ = adjusted R-square.

*CAARMS* comprehensive assessment of at-risk mental states, *SANS* scale for the assessment of negative symptoms, *MADRS* Montgomery-Åsberg Depression Rating Scale, *PSP* personal and social performance scale, *AQoL-8D* assessment of quality of life.

### Relationship between self-perceived cognitive deficits and adherence to cognitive remediation

As depicted in Table 3, no significant associations were found between the baseline MIC-SR total score and number of neurocognitive training hours, number of CR sessions attended, drop-out of therapy, or change scores on the BACS composite score pre/post-treatment.

**Table 3:** Spearman’s correlations between Measure of Insight into Cognition—Self Report (MIC-SR) total score and adherence to and outcome of a cognitive remediation (CR) intervention in UHR individuals (*N* = 52).

	Neurocognitive training hours	CR sessions attended	Drop-out CR	BACS composite change scores
MIC-SR	rho = 0.002	rho = −0.029	rho = 0.177	rho = −0.010
	*p* = 0.988	*p* = 0.842	*p* = 0.215	*p* = 0.949

## Discussion

In keeping with previous literature in psychosis^11–14^ and other psychiatric disorders such as depression^24^ and bipolar disorder^25^, we did not find a significant association between subjective and objectively measured cognitive deficits in UHR individuals. This discrepancy could reflect general difficulties in self-evaluation in psychotic disorders^26^, or it could reflect the notion that subjectively reported, and laboratory based assessment of neuropsychological functioning may measure different aspect of cognition, with self-reported measures having greater ecological validity as they tap cognitive difficulties as the appear in the individuals daily life^27–29^. This potential of measures of subjective and objective cognitive deficits tapping different aspects of cognition is further reflected in the finding of subjective cognitive deficits being unrelated to estimated IQ, while objective cognitive deficits (BACS composite score) were significantly related. Furthermore, our study found UHR individuals to report self-perceived cognitive deficits of a magnitude exceeding that of patients with established psychosis (mean MIC-SR total score in UHR = 21/SD = 7, schizophrenia = 14.0/SD = 9.7 and 13.9/SD = 9.40)^12,14^. This is rather surprising as UHR individuals in general display performance-based cognitive deficits of a smaller magnitude than patients with schizophrenia^7^. Additionally, a little over one-third (39%) of our sample was not found to be cognitively impaired on the performance-based neurocognition measure but still reported self-perceived cognitive impairments in the same range as those with established cognitive deficits. This contrasts some studies in schizophrenia finding that a substantial number of patients do not have an awareness of cognitive deficits even in the presence of objectively measured deficits^12,13,30^. This discrepancy indicates that while patients with schizophrenia may underestimate their level of cognitive deficits, UHR individuals may overestimate their cognitive difficulties. Speculating, this could potentially cause UHR individuals to experience defeatist beliefs and lead to daily-life functional impairments^31,32^. Adding weight to this hypothesis is our cross-sectional finding of self-perceived cognitive difficulties being adversely associated with overall functioning and quality of life explaining 7% and 22% of the variance on these functioning measures, respectively. This warrants a need for future studies investigating whether potential mediators such as defeatist beliefs or perceived competence may mediate the relationship between self-perceived cognitive impairments and functional decrements. Furthermore, it suggests a need for assessing both self-perceived and actual cognitive deficits in UHR groups. In cases of discrepancy between these domains, psychoeducation on cognitive deficits and a focus on cognitive strengths and resilience, could be instituted. While this is, to our knowledge, the first study to elucidate on subjective neurocognitive deficits in a UHR population, a previous study has assessed self-perceived social cognitive impairments in a UHR sample finding a correlation between subjectively experienced cognitive deficits and decrements in role functioning^33^ which further supports the existence of a link between self-perceived cognitive deficits and functioning in UHR.

Regarding the relationship between self-perceived cognitive deficits and psychopathology, we found a significant association with attenuated psychotic symptoms, but not depressive or negative symptoms. This contrast findings from patients with schizophrenia of significant associations between self-perceived and clinician-rated cognitive impairments and depressive, but not positive (or negative) symptoms^12,30^. These findings from schizophrenia samples have indicated that those with higher depressive scores may have greater awareness of cognitive dysfunctions which echoes reports of correlations between depressive symptoms and better insight into psychosis^34^. Findings are, however, mixed, as another study reported less severe depressive symptoms in schizophrenia samples with better insight into neurocognitive deficits^13^. Our finding of an association between self-perceived cognitive impairments and attenuated psychotic symptoms are in accordance with a previous report from a UHR sample, albeit this relationship was observed between self-perceived social cognitive deficits and attenuated psychotic symptoms^33^. It does, however, contrasts with the essentially weak relationship found between performance-based neurocognition and attenuated- and fully psychotic symptoms^35–37^. Hypothesizing, our finding may suggest that some UHR individuals may be inclined to perceive daily life cognitive impairments in areas such as attention, memory, and problem-solving influence level of subthreshold psychotic symptoms (which includes disorganization), or vice versa.

Lastly, this study aimed to elucidate on the relationship between baseline subjective cognitive deficits and adherence to, and benefits from, a cognitive remediation intervention in UHR. We did not find that self-perceived cognitive difficulties were significantly associated with attendance, adherence, or engagement in a cognitive remediation intervention. Neither did it significantly associate with change scores on the global neurocognitive outcome. In previous reports from patients with schizophrenia, poor insight into neurocognitive deficits has not been adversely related to cognitive remediation attendance, treatment satisfaction^13^, or cognitive benefits^13,14^, although it may impact engagement in treatment via its interaction with perceived competency, a motivational construct that promotes treatment engagement^14^.

While being the hitherto largest RCT on cognitive remediation in the UHR population, our study is limited by a relatively small sample completing the MIC-SR (*N* = 52). Furthermore, the RCT study was not designed, nor potentially adequately powered, to address the current research question, which warrants a need for future well-powered studies investigating self-perceived cognitive deficits in response to a cognitive remediation intervention in UHR states. Finally, the study enrolled adults at UHR for psychosis, and while this is similar to other large-scale UHR research studies^38^ the age range is somewhat older than many other UHR studies^39–43^, which may limit the generalizability of the findings to the entire UHR population.

To conclude, our study extends previous findings from schizophrenia and other psychiatric populations of a lack of relationship between objectively and subjectively measured neurocognitive deficits to the population of individuals at UHR for psychosis. Also, our results suggest that UHR individuals may overestimate their level of cognitive deficits indicating this to be a target of psychoeducational approaches. Additionally, we found self-perceived cognitive deficits to associate with overall functioning and quality of life which, if replicated, point towards a target for pro-functional intervention approaches. Future research would benefit from investigating whether psychological aspects such as defeatist beliefs and perceived competence could serve as potential mediator between self-perceived cognitive deficits and functional outcome and benefits from a cognitive remediation intervention in UHR. Finally, we did not find baseline self-perceived cognitive deficits to relate to engagement in- or outcome of cognitive remediation in UHR indicating that subjectively experienced cognitive deficits do not seem to constitute a suitable clinical selection-based criterion of whether individuals should be offered a cognitive remediation intervention.

## Methods

### Study design

A detailed description of the original study can be found in ref. ^44^. Briefly described, participants were recruited to the randomized clinical, FOCUS trial from the psychiatric in- and outpatient facilities in the greater catchment area of Copenhagen, Denmark from April 2014 to December 2017. On completion of baseline assessments, the participants were randomly assigned to either 20-weeks of cognitive remediation as an add on to treatment as usual (TAU + CR) or to treatment as usual (TAU). The cognitive remediation intervention comprised 2 h of group training (1 h of neurocognitive training, with subsequent 15 min of bridging session, and 1 h of social cognitive training) once a week for a total of 20 weeks. The cognitive remediation targeted both neuro- and social-cognition using the manualized, evidence-based treatments; the Neuropsychological Educational Approach to Cognitive Remediation (NEAR)^45^ and the Social Cognition and Interaction Training (SCIT) manual^46^. Supplementary to the group training, the participants received 12 individual sessions to personalize and maximize the transfer of the effect of the cognitive training to the participants’ daily lives. In addition, participants were encouraged to conduct a minimum of 1-h weekly of home-based neurocognitive training. The neurocognitive training was scaled to comprise 40 h of neurocognitive remediation, expecting that each participant attended the 20-group sessions and did the recommended 1-h weekly of home-based training.

The study protocol was approved by the Committee on Health Research Ethics of the Capital Region Denmark (study: H-6-2013-015). All participants provided written informed consent prior to inclusion in the study. Trial registration ClinicalTrial.gov identifier: NCT02098408.

### Participants

The sample consisted of 146 help-seeking individuals aged 18–40 years, who met one or more UHR criteria according to the Comprehensive Assessment of At-Risk Mental States^47^: attenuated psychotic symptom group; brief limited intermittent psychotic symptoms group; and/or trait and vulnerability group along with a significant drop in functioning or sustained low functioning for the past year.

### Assessments

The demographic information assessed in the study comprised age, years of education, and estimation of current IQ, with the latter assessed using four subtests from the Wechsler Adult Intelligence Scale - Third Edition (WAIS-III)^48^: Vocabulary, Similarities, Block Design, and Matrix Reasoning.

### Self-perceived cognitive deficits

The Measure of Insight into Cognition—Self Report (MIC-SR)^12,49^ was used to assess self-perceived cognitive impairments. The MIC-SR comprises 12 items on daily life difficulties with memory, attention, and problem solving. Each of the 12 items are given a score of 0–3 corresponding to experiencing cognitive difficulties “never” (0), “once a week or less” (1), “twice a week” (2), or “almost daily” (3). A total score can be extracted summing the ranges from 0 to 36. An average frequency score can be computed dividing the total score by 12. The MIC-SR has proven reliability and validity in patients with schizophrenia^49^.

### Performance-based cognitive performance

Performance-based cognition was indexed with the Brief Assessment of Cognition in Schizophrenia (BACS) battery^50^. The BACS composite score derives from six subtests of the domains of verbal learning and memory, speed of processing and executive functions.

### Clinical symptoms and functioning

Symptoms were assessed with three instruments; the CAARMS, providing a level of attenuated psychotic symptom, by weighing the intensity of symptom scores by their frequency to form a CAARMS composite score^51,52^; the Scale for the Assessment of Negative Symptoms (SANS)^53^ yielding the level of negative symptoms using the SANS global score; and the Montgomery-Åsberg Depression Rating Scale (MADRS)^54^ yielding level of depressive symptoms. Functioning was measured with two instruments capturing different aspects of functioning: Overall functioning was measured using the Personal and Social Performance Scale (PSP)^55^. This is a composite measure of functioning in the areas of occupational functioning, social functioning, and self-care. Subjective quality of life was reported with the Assessment of Quality of Life (AQoL-8D)^56^. The clinical assessors were all psychologists and medical doctors, that had received extensive training in using the instruments and conducted regular inter-rater reliability ratings on selected outcomes. Cognitive tests were conducted by psychologist students that had received comprehensive training and were supervised regularly by a senior psychologist.

### Statistical analysis

Analyses were performed using SPSS version 25.0. Baseline descriptive statistics were reported by means and standard deviations. One-way analyses of variance (ANOVA) was used to compare the group displaying performance-based cognitive deficits with the group that did not on the MIC-SR. Stratification of the sample was based on whether or not the participants displayed a BACS composite score of ≤−1.35, as previous findings indicate this to be a relevant cut-off to determine borderline to impaired cognition^12^. Univariate linear regression analyses were run to investigate the association between the dependent variable of MIC-SR and the independent variables of clinical symptoms (CAARMS, SANS, MADRS) and functioning (PSP and AQoL-8D). Bivariate correlations, using a Spearman’s correlation coefficient with a two-tailed significance test, were run to investigate the correlations between MIC-SR total score and BACS composite score at baseline and as difference score pre/post-treatment along with indices of adherence to the cognitive remediation intervention. Significance levels were set to *p* < 0.05.

### Reporting summary

Further information on experimental design is available in the Nature Research Reporting Summary linked to this paper.

## Supplementary information

Reporting Summary Checklist

## Data Availability

The data that support the findings of this study are available from the corresponding author upon reasonable request.
